# Effects of the COVID-19 pandemic on the mental health of rehabilitation area professionals: A systematic review

**DOI:** 10.3389/fpubh.2022.1085820

**Published:** 2022-12-08

**Authors:** Sandra Bohórquez-Blanco, Regina Allande-Cussó, Cristina Martín-López, Juan Gómez-Salgado, Juan Jesús García-Iglesias, Javier Fagundo-Rivera, Carlos Ruiz-Frutos

**Affiliations:** ^1^Physiotherapy School, Industrial University of Santander, Bucaramanga, Santander, Colombia; ^2^Labour Risks Prevention Master, Faculty of Labour Sciences, University of Huelva, Huelva, Spain; ^3^Department of Nursing, Nursing, Physiotherapy, and Podiatry School, University of Seville, Seville, Spain; ^4^Rehabilitation Area, Riotinto Hospital, Huelva, Spain; ^5^Department of Sociology, Social Work and Public Health, Faculty of Labour Sciences, University of Huelva, Huelva, Spain; ^6^Safety and Health Postgraduate Programme, Universidad Espíritu Santo, Guayaquil, Ecuador; ^7^Centro Universitario de Enfermería Cruz Roja, University of Seville, Seville, Spain

**Keywords:** health professionals, physiotherapist, rehabilitation, mental health, psychological stress, anxiety, depression, COVID-19

## Abstract

**Background:**

The role of the physiotherapist is vital in the recovery of post-COVID-19 patients, but fear of contagion is a possible feeling among healthcare professionals. The objective of this study is to assess the mental health effects that COVID-19 has had on healthcare workers, including rehabilitation care, in times of pandemic.

**Methods:**

A systematic review was conducted using the PRISMA format in the Pubmed, SCOPUS, and Web of Science databases between July and September 2022. Keywords included were “healthcare providers,” “COVID-19,” “Mental Health,” and “Psychological Distress.” Methodological quality was assessed using the Joanna Briggs Institute critical appraisal tools.

**Results:**

A total of 14 studies were included in this review. The study population was healthcare professionals including the rehabilitation services. In total, 4 studies reported exclusively on anxiety and stress levels in physiotherapists providing care during the pandemic.

**Conclusions:**

The mental health of healthcare professionals has been compromised during the pandemic. However, initially, research was only focused on physicians and nurses, so the need arises to include those professionals, such as physiotherapists, who are also in direct contact with COVID-19 patients.

**Systematic review registration:**

https://www.crd.york.ac.uk/prospero/display_record.php?RecordID=367664, identifier: CRD42022367664.

## Background

COVID-19 is a disease caused by Severe Acute Respiratory Syndrome Coronavirus 2 (SARS-CoV-2) (1), so defined because of its similarities to the 2003 SARS-CoV virus, with which it shares RNA characteristics but can cause both mild and severe respiratory infections. As the pandemic has progressed, the World Health Organization (WHO) has been regularly updating the classifications of SARS-CoV-2 by considering its phenotypic characteristics, degree of complexity, mode of manifestation, and geographical distribution (1). According to WHO, those most at risk of severe SARS are people over 60 years of age and those with comorbidities or pre-existing diseases, such as people with diabetes, obesity, cancer, or hypertension, among others. However, any person, regardless of age or health status, can develop complications, as can be seen by the high mortality rates, with a total of 6,547,162 deaths and 618,144,676 diagnosed cases worldwide as of October 2022, according to the Johns Hopkins Coronavirus Resource Center (2). The impact on the health system is massive as well as on Physical Rehabilitation Medicine services throughout the countries (3).

For COVID-19 to be declared a pandemic in March 2020, it had to show alarming levels of spread and severity affecting a large number of people, as well as outbreaks in more than one continent. Some of the consequences of SARS-CoV-2 implied limitations in participation and restrictions in access to different care spaces and services. Many of the diagnosed patients and others with different pathologies began to receive care through online appointments, leaving face-to-face consultations for more serious cases in order to combat the onslaught of the disease and its spread (4, 5). In addition, borders and some facilities were closed to help mitigating the psychological, environmental, and economic effects of COVID-19 (6, 7).

COVID-19 also has a serious impact on people's mental health (8). Psychological stress, including depression and anxiety, has been reported by healthcare workers with high frequency during the time of the pandemic (8, 9). Several factors increase the risk of mental health issues, including exposure to social, economic, geopolitical, and environmental circumstances. Mental health risks and protective factors are found in society at different scales, although, the most vulnerable people have taken the greatest impact (10). Global threats increase the risk of mental illness, including disease outbreaks, humanitarian emergencies, and forced displacement, among others (8, 11). But there are more vulnerable groups of professionals which had been affected by this pandemic, in example, those who worked in nursing homes, where access to protection measures was scarce and consequences went lethal both for professionals and residents (3). The pandemic has left great changes in its waves, with an impact on the mental health of people. Therefore, it is important to design and adopt protection strategies for the mental health of health professionals, as well as the early diagnosis of possible mental health problems (10).

The Job Demands-Resources (JD-R) model, in contrast to the theories of job design and job stress, highlights the role of job stressors, being used to predict burnout, engagement, and additionally, to identify the consequences of sickness absenteeism and job performance. With the JD-R model, it is possible to explain, understand, and predict employees' wellbeing. According to the theory, work environments can be divided into job demands and job resources, and this can be applied to all occupations. However, there are job demands and job resources specifically relevant to each occupation or profession (11). Job demands refer to those physical, psychological, organizational, or social aspects (e.g., work pressure, emotional demands, burnout) of work that require sustained effort, while job resources refer to those variants that can reduce the demands of work (e.g., social support, autonomy, development opportunities, organizational climate, commitment, etc.). In this sense, job resources are necessary to cope with job demands. Therefore, the interventions to be undertaken at the company level are both personal and organizational, applied in the redesign of the job, the job position, and/or by providing training resources that meet the objective of the intervention (11, 12).

According to a systematic review, some of the risk factors most associated with psychological distress during the COVID-19 pandemic were being female, from lower socioeconomic status (lower income, lower level of education, and unemployment), belonging to rural areas, and those at higher risk of COVID-19 infection (healthcare professionals, older people, or people with comorbidities). These population groups showed a higher prevalence of suffering episodes of depression and anxiety compared to other groups (12). In fact, during the first months of the pandemic, between 70 and 90% of health workers who were exposed to high risks, triggered various health problems, including stress, anguish, anxiety, fear, irritability, among others. This led to potentiate negative effects on the mental health of health workers, including the development of post-traumatic stress as part of a long-term problem resulting from this pressure (13, 14).

There is the fact that research during this time of pandemic has yielded significant outcomes in different areas, being one of them healthcare professionals. However, it is understood that “those in the front line” are only physicians and nurses (13), leaving aside other key occupations in the recovery process of patients. For this reason, generating knowledge and evidence from other healthcare professions such as rehabilitation professionals is crucial to understand the effects of the pandemic (1).

Healthcare professionals play a major role in the care and contact with people with COVID-19, many of them being part of the first line of defense against the virus (13). They may also be afraid of infecting their family and friends, suffering from social discrimination, and experiencing increased work stress due to the high demand of patients in care, even leading to, in some cases, a decrease in the quality of care (15, 16). For all these reasons, the healthcare personnel may experience emotional disorders (anxiety, fear, depression), sleep problems, and even post-traumatic stress in those who have participated in previous outbreaks. Therefore, the physical and mental wellbeing of healthcare staff is compromised, and its preservation may be essential to combat the effects that COVID-19 leaves in its wake (16).

Due to the impact of the pandemic on all health services, a restructuring of rehabilitation services was initiated as Physical Therapy areas were transformed into temporary hospitalization rooms. In fact, the Spanish Society of Physical Medicine and Rehabilitation (17), published in 2020 recommendations in relation to health care and home restrictions, considering the consequences of the pandemic on population's health and leading health professionals (including rehabilitation services) to take on new challenges in patient care, appropriate treatment, and protocols to prevent the spread of the virus (3). That was a primary concern that required all rehabilitation professionals to participate in a comprehensive assessment in search of optimal care measures with focus on the patient's recovery, but also in controlling the spread of COVID-19. With this publication, it was reassured the necessity of knowing about the psychological impact of the pandemic on rehabilitation professionals, including, as part of future research, relating or reviewing differences with respect to other similar pandemics (e.g., SARS-CoV-1 and MERS) (14).

The aim of this study was to assess the effects that COVID-19 has on the mental health, i.e., the psychological distress, of healthcare workers of the rehabilitation services when caring for patients in times of pandemic.

## Methods

### Study design

A systematic review was conducted following the guidelines of the PRISMA statement (18) (Preferred Reporting Items for Systematic reviews and Meta-Analyses). For this purpose, the authors used a protocol to carry out this systematic review, which was registered in the International Prospective Register for Systematic Reviews (CRD42022367664).

### Databases and search strategy

The search was carried out in the following electronic databases: Pubmed, SCOPUS, and Web of Science. It was based on the key words provided by the research question that followed the PICOT strategy (Table 1). Gray literature resources were not assessed.

**Table 1:** PICO format: keywords (rehabilitation and COVID-19, Spain, 2022).

**Population**	**Physiotherapist**
Intervention	To assess mental health (depression, anxiety, stress, and fear)
Comparator	Mental health levels of other healthcare professionals
Outcomes	Prevalence of cases of people with depression, anxiety, stress, and fear; comparison of levels before and during the COVID-19 pandemic, predisposing vs protective factors, differences between countries, comparison according to type of profession/service.
Time period	During the COVID-19 pandemic
Research question	*How has the COVID-19 pandemic affected anxiety and fear levels in rehabilitation professionals?*

Following these keywords, the Medical Subject Headings (MeSH) thesaurus was consulted, yielding the descriptors health personnel and physiotherapists, mental health, physiological stress, anxiety, depression, and COVID-19. In order to enlarge the scope of the search, synonymous terms were used to complete the search based on the Medical Subject Headings (MeSH) descriptors (Table 2), linked by the Boolean operators AND and OR.

**Table 2:** Terms used in the search (Rehabilitation and COVID-19, Spain, 2022).

**MeSH**	**Terms**
Health personnel	Healthcare Professionals *OR* Healthcare Workers *OR* Healthcare Providers *OR* Professional Health Care
Physical therapists	*Physical Therapist OR therapist OR physiotherapist*
COVID-19	Coronavirus 2019 *OR* 2019-Ncov *OR* Cov-19 *OR* Coronavirus Disease-19
Mental health	Mental health
Psychological distress	Stress
Anxiety	Anxiety
Fear	Fear
Depression	Depression

Table 3 shows the search strategy used on 27 July 2022 for each of the above databases during the search process.

**Table 3:** Search strategy used by database (rehabilitation and COVID-19, Spain, 2022).

**Database**	**Search strategy**	**Results**	**Selected**
Pubmed	((depression[Title/Abstract] OR anxiety[Title/Abstract] OR stress[Title/Abstract] OR fear[Title/Abstract] OR mental health[Title/Abstract]) AND (COVID-19[Title/Abstract])) AND (physiotherap*[Title/Abstract])	86	17
	Filters: publication date years 2020–2022		
Scopus	(TITLE-ABS-KEY (depression OR anxiety OR stress OR fear OR mental AND health) AND TITLE-ABS-KEY (COVID-19) AND TITLE-ABS-KEY (physiotherap*)) AND PUBYEAR >2019	247	22
Web of Science	TS = (Depression OR anxiety OR stress OR fear OR mental health (Topic) AND COVID-19 (Topic) AND physiotherap* (Topic)) Refined by: years 2020–2022 and type of document (ARTICLE) and search in: HUMANS	148	9
Google scholar	Items identified through other resources	5	2
Total	490	50/14^a^	

a After eliminating duplicates.

### Selection criteria

Original articles, including meta-analyses, systematic reviews, cohorts, cross-sectional, and case-control studies published in English and Spanish were included in this review.

The following criteria were used for the selection of articles:

#### Inclusion criteria

- Original articles published in English and Spanish.
- Articles published from 2020 to date.
- Type: original articles, meta-analysis, case reports.
- Articles measuring any of the following values and/or effects: level of depression, level of stress and level of anxiety, number of cases of professionals with depression, stress and/or anxiety, comparison of levels before vs. during the COVID-19 pandemic, and comparison according to country or type of profession/service.

#### Exclusion criteria

- Studies that did not meet the previously established inclusion criteria, that did not answer the research question, or that were not related to the objective of the review.
- Studies of low scientific-technical quality after applying the quality assessment tool.
- Study population other than healthcare professionals and which did not include rehabilitation professionals.
- Typology: opinion articles, commentaries, editorials and letters to the editor/head, and quasi-experimental.

### Data collection and extraction

Initially, two researchers independently carried out the searches, as set out in the search strategy for each of the chosen databases. Subsequently, one researcher eliminated duplicate articles and those that did not meet the previous criteria, and finally included studies accordingly, after reading the titles and abstracts. Subsequently, one author reviewed the full text of the potential studies for the review and made the decision to include or exclude them. Discrepancies were resolved by the first two authors.

### Assessment of methodological quality

The methodological quality of the selected studies was determined using the critical appraisal tools of the Joanna Briggs Institute (JBI) of the University of Adelaide (19). The purpose of this tool is to assess the methodological quality of a study and to determine the extent to which a study has excluded or minimized the possibility of bias in its design, conduct, and/or analysis. The versions for quantitative cross-sectional studies (19) (8 items), the JBI checklist for analytical cohort studies (20) (11 items), and for qualitative studies (21) (10 items) were used, setting the cut-off point at 6 for acceptance for inclusion in this review (see Supplementary Tables S1–S3 in Supplementary material).

Table 4 shows the characteristics of each of the 14 final articles for this review, and it is based on the Iberoamerican Cochrane Centre Handbook guidelines (22). These characteristics were categorized by authors and year of publication, geographical context, objective, type of study, participants, measurement instrument(s), and main findings; in addition, the results of the JBI critical appraisal tool were added.

**Table 4:** Characteristics of the studies included in the systematic review (rehabilitation and COVID-19, Spain, 2022).

**References**	**Context**	**Study objective**	**Type of study**	**Participants**	**Methods**	**Main findings**	**Quality**
Alnaser et al. (23)	Kuwait (Western Asia)	To examine the level of anxiety among occupational therapists (OTs) and physiotherapists (PTs) who have interacted with patients during the COVID-19 pandemic	Quantitative cross-sectional study	Occupational therapists and physiotherapists in public hospitals (*n* = 98)	GAD-7 PHQ-15	GAD-7 and PHQ-15 were positively correlated (*p* < 0.000). Likewise, an association was shown between anxiety levels and somatic symptoms in both tests (*p* < 0.000). The final overall GAD-7 score was (9.21 ± 5.63), showing 27% of participants with no anxiety and 21% with severe anxiety. Significant differences were obtained between occupational and physical therapists for GAD-7 scores (*p* = 0.026), with PTs having higher anxiety levels than occupational therapists (μ = 8.13 ± 5.49). Additionally, therapists residing with their parents showed higher levels of anxiety vs. those residing without their parents (*p* = 0.013), as did those working in neurology compared to the other services (pediatrics and orthopedics)	6/8
Aly et al. (24)	Egypt	To assess perceived stress, anxiety, and depression among healthcare workers facing the COVID-19 pandemic in Egypt	Quantitative cross-sectional study	Physicians, physiotherapists, nurses, and others (*n* = 316)	GAD-7 PSS-10 PHQ-9	98.5% of the sample showed moderate to severe stress levels, 90.5% showed some degree of anxiety, and 80% showed varying degrees of depression, ranging from mild to severe. About 87% of the participants suffered from all 3 disorders (stress, anxiety, and depression), and only 3.5% suffered from only one. The three mental health disorders assessed showed no statistically significant differences between the different socio-demographic characteristics (age, sex, marital status) (*p* > 0.05)	7/8
Chatzittofis et al. (25)	Republic of Cyprus (Mediterranean)	To assess the mental distress of healthcare workers during the COVID-19 pandemic in Cyprus, in particular the presence of symptoms of post-traumatic stress disorder, depression, and anxiety	Quantitative cross-sectional study	Nurses, physicians, physiotherapists, and others (*n* = 428)	PHQ-9 IES-R PSS-10	Older age in men was a statistically significant variable associated with reduced scores on the PHQ-9 (*p* = 0.003) and the IES-R scale (*p* = 0.005). A history of depression was associated with increased mental health disorders and depressive symptoms during the pandemic (*p* = 0.02); however, a personal history of anxiety was not associated with mental health disorders or intensity of depressive symptoms (*p* = 1.1). In addition, an inverse association was observed between years of work experience and PHQ-9 score (*p* = 2.6)	6/8
de Sire et al. (26)	Calabria (Italy)	To assess the correlation between work environment factors and psychological distress in a cohort of physiotherapists working in hospitals in southern Italy during the COVID-19 pandemic	Quantitative cross-sectional study	Physiotherapists in clinics with COVID-19 patients (*n* = 80)	Online local questionnaire C.A.L.A.B.R.I.A (nine questions)	Physiotherapists working in the public sector reported higher confidence in their skills (aτ = −0.32, *p* < 0.01) and their employers worked harder to ensure good and safe conditions (aτ = −0.48, *p* < 0.001)	6/8
Jácome et al. (27)	Porto (Portugal)	To describe burnout among physiotherapists working in Portugal and to analyze possible predictors during the COVID-19 pandemic	Cross-sectional study	Physiotherapists working during the COVID-19 pandemic (*n* = 511)	CBI. Resilience Scale DASS-21 Satisfaction with Life Scale.	42% of participants reported work-related distress and 25% reported patient-related distress. 55% of patients reported moderate levels of resilience and only 18% indicated levels of stress, anxiety, and depression. Significant differences were found in scores for personal (*p* = 0.001), work-related (*p* = 0.043), and anxiety levels (*p* = 0.019) of burnout between physiotherapists who directly cared for COVID-19 patients and those who did not. Correlations between measures of burnout, resilience, depression, anxiety, and stress were all statistically significant (*p* < 0.001)	6/8
Medeiros et al. (28)	Fortaleza (Brazil)	To document the prevalence of each burnout domain and the factors associated with these domains during the COVID-19 outbreak.	Quantitative cross-sectional study	Physicians, nurses, auxiliary nurses, and physiotherapists (*n* = 265)	-MBI-HSS	48.6% showed high levels of emotional exhaustion and almost 1/3 (29.4%) of them, showed high levels of depersonalization. 18.1% showed low levels of professional efficacy	6/8
						The determinants of burnout due to depersonalization were age <33 years (OR 2.03; 95% confidence interval, CI 1.15–3.56; *p* = 0.01) and being female (OR 0.33; 95% CI 0.18–0.62; *p* = 0.01). Increased workload was associated with emotional exhaustion (OR 1.89, 95% CI 1.04–3.58, *p* = 0.030).	
Pigati et al. (29)	Sao Paulo (Brazil)	To investigate the levels of stress, depression and anxiety, event impact, resilience, and the determinants/modulators of these responses in physiotherapists working or not in contact with patients with COVID-19	Quantitative cross-sectional study	Physiotherapists (*n* = 519)	IES-R DASS-21	Physiotherapists with low resilience showed significantly higher depression, anxiety, stress, and event impact scores compared to the high resilience group (*p* < 0.001). In addition, working with COVID-19 patients increased levels of depression, anxiety, stress, and event impact compared to the non-COVID-19 group (*p* < 0.001). These responses were modulated by age, sex, number of absences from work, and whether these took place or not	8/8
Syamlan et al. (30)	Indonesia	To explore mental health status and health-related quality of life (HRQoL), and to identify determinants, in healthcare workers in Indonesia	Quantitative cross-sectional study	Nurses, physicians, physiotherapists, and others (*n* = 502)	HQol SF12V2 DASS-21	Of the total respondents, 29.4% experienced depression, 44.9% anxiety, and 31.8% reported stress. In addition, depression, anxiety, and stress were more prevalent in women (34.7, 50.6, and 44.9%, respectively). Work during the COVID-19 pandemic was found to be statistically significant (*p* = 0.015)	7/8
Szwamel et al. (31)	Poland	(17) To analyse the burnout phenomenon, the level of anxiety, depression, and quality of life among healthcare workers in times of the COVID-19 pandemic. (1) To establish the factors that significantly determine the level of occupational burnout in this group.	Quantitative cross-sectional study	Nurses, physiotherapists, physicians, and others (*n* = 356)	MBI HADS WHOQOL BREF	71.63% showed high and moderate levels of emotional exhaustion during the pandemic, 71.43% reported low and moderate levels of job satisfaction, while 40.85% showed high and moderate levels of depersonalization. In addition, 62.57% showed borderline anxiety disorders and 83% (*n* = 193) suffered from depression. Emotional exhaustion seemed to be much higher in nurses and other healthcare professionals than in physiotherapists (*p* = 0.023)	8/8
Yang et al. (32)	South Korea	To investigate mental health burden by COVID-19, including stress and anxiety levels, in physiotherapists	Quantitative cross-sectional study	University hospital physiotherapists (*n* = 73)	GAD-7 PHQ-9	21 out of 73 physiotherapists showed anxiety (GAD-7 ≥ 5) and 12 out of 73 physiotherapists (18.5%) showed depression (PHQ-9 ≥ 10). The results revealed that physiotherapists who lived with an infant or child ≤6 years or a person ≥65 years had a significantly higher risk of suffering from anxiety (*p* = 0.014). If a physiotherapist had an infant or child ≤6 years, the risk of anxiety was significantly increased, reaching 6.72 times higher than for those who did not have a child ≤6 years (*p* = 0.007)	7/8
Farì et al. (33)	Southern Italy	To assess the impact of COVID-19 on the mental health burden of Italian healthcare workers, comparing their condition with that prior to the emergency	Quantitative, analytical, retrospective cohort study	Physicians, nurses, and physiotherapists (*N* = 68)	PHQ-9 GAD-7 MBI	50% of the assessed professionals scored above the cut-off point for burnout during the COVID-19 emergency. Moreover, it increased by 17% compared to the levels before the pandemic (*p* < 0.0001). The PHQ-9 scale showed statistically significant differences between before and during the pandemic (*p* < 0.0001), and anxiety levels tripled during the pandemic (*p* < 0.0001). Differences on the PHQ-9 were found in women being more exposed to anxiety (*p* = 0.040).	8/11
Jeleff et al. (34)	Vienna (Austria)	To address the structural determinants and the physical, mental, emotional, and professional challenges of HCWs during the COVID-19 pandemic	Exploratory qualitative study	Physicians, nurses, physiotherapists, and others (n=30)	Semi-structured interviews (30 min)	There was a lack of preparedness (shortage of personal protective equipment and critical patient conditions), structural conditions that could be improved (understaffing and overload), and concerns about the physical and mental health of healthcare workers (stigma and avoidance behavior of colleagues)	7/10
Palacios-Ceña et al. (35)	Madrid (Spain)	To describe and explore the experiences and perspectives of physiotherapists working in public hospitals in Madrid, Spain, during the COVID-19 pandemic	Exploratory qualitative study	Physiotherapists (*n* = 30)	Interviews Inductive thematic analysis	3,912 codes and 13 categories were identified, resulting in 3 topics. As the COVID-19 infection spread dramatically, hospitals became contaminated and overwhelmed, and all floors became COVID-19 rooms (call of duty). Secondly, every day, therapists received ‘the war report' and orders, complied with personal protective equipment requirements, and faced fear (working in wartime). Finally, working during the pandemic had an impact on the therapists' families and the information shared with them (when I get home)	9/10
Palacios-Ceña et al. (36)	Madrid (Spain)	To explore the emotional experience and feelings of physiotherapists working in the frontline in public health hospitals in Madrid (Spain) during the first outbreak of COVID-19	Exploratory qualitative study	Physiotherapists from rehabilitation services in public hospitals (*n* = 30)	Interviews Inductive thematic analysis	2,135 codes and nine categories were identified and three topics emerged to describe emotional experiences and feelings. Firstly, related with negative and positive critical events (Critical Events). Secondly, with emotions, feelings, and coping strategies (Emotional Rollercoaster). Finally, on the conclusions of the COVID-19 outbreak experience, with the meaning of the COVID-19 outbreak from a personal and professional perspective (Last words)	8/10

GAD-7, Generalized Anxiety Disorder (range 0–21; scale 0–3; Minimal anxiety 0–4/Mild anxiety 5–9/Moderate anxiety 10–14/Severe anxiety ≥ 15); PHQ-9, Patient Health Questionnaire depression module (scale 0–3, range 0–27; None-Minimal 0–4/Mild 5–9/Moderate 10–14/Moderately severe 15–19/Severe 20–27); IES-R22, Impact of Event Scale-Revised (range, 0–88); CBI, Copenhagen Burnout Inventory; DASS-21, Depression Anxiety Stress Scales (Depression, Normal 0–9/ Mild 10–13/Moderate 14–20/Severe 21–27/Extremely severe ≥ 28); MBI, Maslach Burnout Inventory; MBI-HSS, Maslach Burnout Inventory, Human Services Survey version; HADS, Hospital Anxiety Depression Scale; PSS-10, Perceived Stress Scale (scores from 0 to 13 “low stress”; from 14 to 26 “moderate stress,” and between 27 and 40 “high perceived stress”; HCW, Healthcare workers; HRQoL, Health-related quality of life; SF12V2, Short Form 12 item (version 2) Health Survey.

## Results

**Figure 1:**
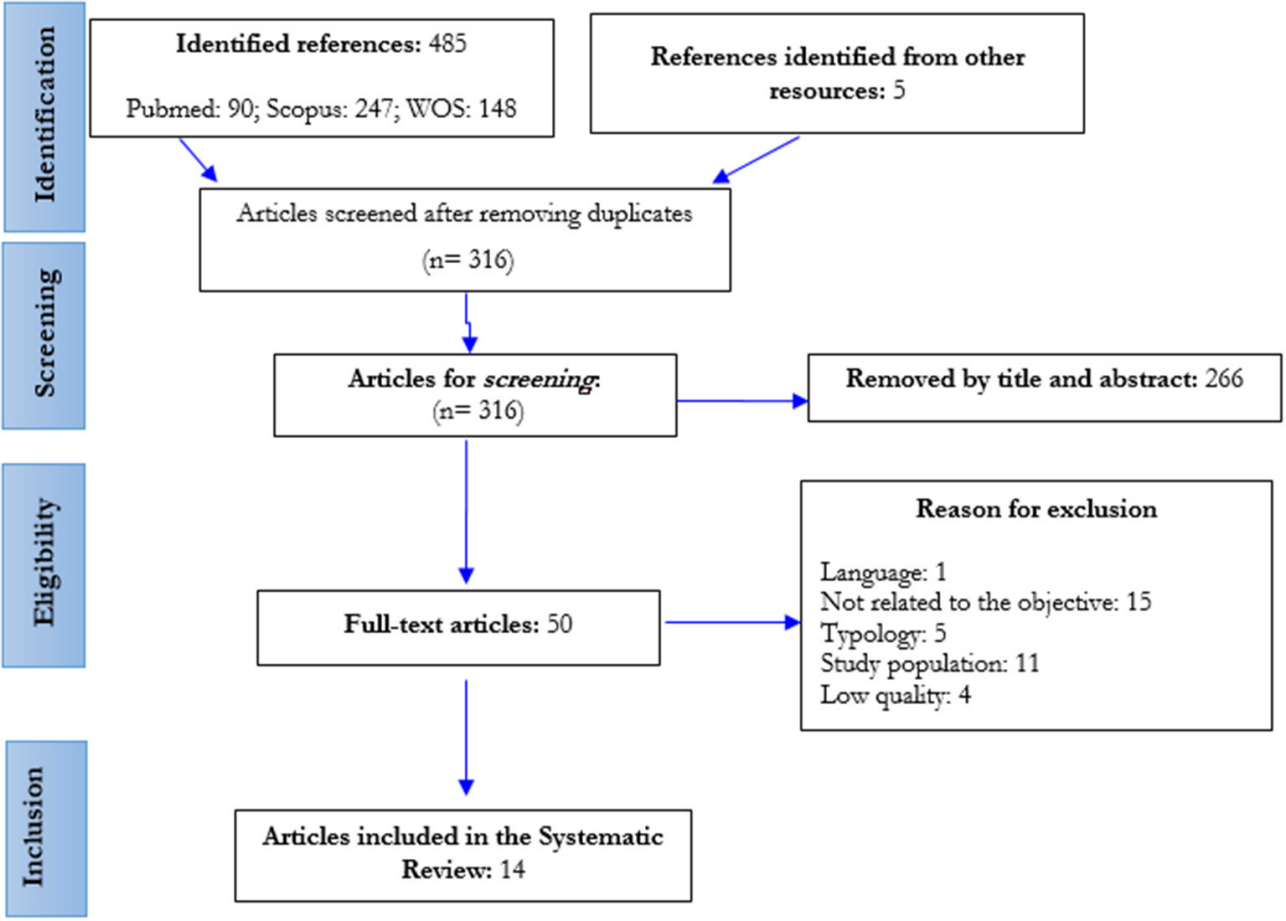
Flowchart of the study selection process (PRISMA) (rehabilitation and COVID-19, Spain, 2022).

The initial search strategies identified a total of 490 references, which were screened according to the topic of this review. A total of 14 articles were finally selected (Figure 1), 11 of which were quantitative (ten cross-sectional and one retrospective cohort) and three qualitative.

There was a variety of countries identified in the studies, among them, two were conducted in Spain, two in Brazil, two in Italy, and one in other countries such as Portugal, Korea, Austria, Indonesia, and Egypt, among others. In relation to the sample, in 6 of the 14 studies included in the review, the sample was exclusively composed by physiotherapists.

The included articles were assessed with the JBI critical appraisal tool, where both quantitative and qualitative studies obtained medium-high scores.

### Level of anxiety

To assess anxiety levels, the different studies used the following scales: GAD-7, DASS-21, and HADS. The Generalized Anxiety Disorder tool (GAD-7) is a self-administered screening test designed to identify probable cases and severity of anxiety. The GAD-7 is used in adults >18 years old and includes 7 items on a Likert-type scale (0 = not at all; 1 = several days; 2 = More than half the days; 3 = Nearly every day). Scoring ranges from 0 to 21, with scores of 5, 10, and 15 set as cut-off points for mild, moderate, and severe anxiety, respectively. Further assessment is recommended when the score is 10 or higher (23).

The level of anxiety can also be assessed with the DASS-21 “Anxiety subscale,” which has been one of the preferred assessing instruments during the COVID-19 pandemic (27, 30, 37, 38). This version consists of a 21-item, four-point Likert questionnaire which includes three self-report subscales designed to measure the negative emotional states of depression, anxiety, and stress (which apply to the participant from not at all to most of the time). Scores for each subscale range from 0 to 21, with higher scores indicating a more negative emotional state.

### Level of depression

The Center for Epidemiologic Studies Depression Scale (CES-D), the Patient Health Questionnaire depression module (PHQ-9), the Depression Anxiety Stress Scales (DASS-21: Depression subscale), the Hamilton Depression Scale (HAMD-17), and the Self-rating Depression Scale (SDS) (39) are available to assess depressive symptoms.

The HADS is one of the scales used in the assessed studies to evaluate depression and anxiety. It had been used before with nursing staff in Poland (31, 39). It originally contained 7 items assessing anxiety and 7 items related to depressive states. After modification, 2 items for irritation and aggression were added. In total, the scale consists of 16 closed questions with 4 possible response options. Each answer can be scored between 0 and 3 points. The categories are distinguished individually for the anxiety and depression subscales (0–7: no disorders; 8–10: borderline state; 11–21: present disorder).

In addition, the level of depression was qualitatively assessed through semi-structured interviews to record and code the emotional experiences of healthcare professionals, including the rehabilitation services, in times of pandemic (34–36).

### Level of stress

The Perceived Stress Scale (PSS-10) consists of 10 questions about feelings and thoughts during the last month. Responses are given for each question on a 5-point scale which ranges from “never” to “very often.” Then, the total is calculated. Scores ranging from 0 to 13 are regarded as low stress; scores from 14 to 26, moderate stress; and between 27 and 40, scores are considered high perceived stress (24, 25).

The 22-item Impact of Events Scale-Revised (IES-R) is used to assess post-traumatic stress symptoms during the past 7 days. Each item is scored from 0 to 4. The total scale score ranges from 0 to 88. Values above the cut-off point of 33 indicate a clinically relevant symptom (25, 29).

### Other data

All studies included questionnaires covering socio-demographic data (age, sex, marital status, and occupation). However, some of them included questions related to the health of the participants (25, 28) and others about the way of working during the pandemic (face-to-face, telework, etc.) (27).

Regarding mental health, some studies (26–29, 34, 36) used the Patient Health Questionnaire (PHQ) to measure the level of somatisation. It is a self-administered version of the PRIME-MD (Primary Care Evaluation of Mental Disorders) diagnostic instrument for common mental disorders (24, 25, 32, 33). The PHQ-15 (23) comprises 15 somatic symptoms from the PHQ. The 15 items are scored on a 3-point Likert scale (0 = does not bother me at all; 1 = bothers me a little; and 2 = bothers me a lot). However, due to cultural sensitivities, two items (question no. 4: menstrual cramps or other problems with your periods and no. 11: pain or problems during intercourse) were removed from the questionnaire and the mPHQ-15 (modified version) with 13 somatic symptoms emerged. The mPHQ-15 total score ranged from 0 to 26 and scores of 3, 18 and 13 were set as cut-off points for mild, moderate, and severe somatisation levels, respectively. The PHQ-15 has demonstrated high reliability and validity for application in clinical and occupational health care settings (23).

The Maslach Burnout Inventory (MBI), on the other hand, assesses the level of burnout. It is composed of 22 items designed to evaluate the three dimensions of burnout: Emotional Exhaustion (nine items); Depersonalization (five items); and Personal Accomplishment (8 items). All MBI items are scored using seven-level frequency ratings, from “never” (=0) to “every day” (=6). Burnout is confirmed by obtaining high scores on the subscales that assess emotional exhaustion (0–54 items) and depersonalization (0–30 items) and low scores on the Personal Accomplishment subscale (0–48 items) (28, 31, 33). Similarly, the Copenhagen Burnout Inventory (CBI) is a scale designed to measure burnout, including 19 items in subscales (personal, work-related, and client-related). All items are scored on a five-point Likert scale (Always/To a very high degree = 100; Often/To a high degree = 75; Sometimes/Somewhat = 50; Seldom/To a low degree = 25; and Never/Almost never/To a very low degree = 0) (27).

The WHOQOL measures health-related quality of life. It was assessed using the Polish version of the abbreviated World Health Organization instrument (WHOQOL BREF). It has 4 domains: D1-Physical; D2-Psychological; D3-Social Relationships; and D4-Environmental, and consists of 26 questions. The respondents rate each aspect on five-point Likert scales. The domain score reflects an individualized perception of each quality-of-life domain, and it is scaled in a positively framed direction: the higher the score, the higher the health-related quality of life (30, 31).

## Discussion

The COVID-19 pandemic has radically led to a change in lifestyle, affecting different aspects (work, family, personal, among others) (40). Newly published research studies recommend the work of the physiotherapist in the recovery of post-COVID-19 patients. However, though mental health is a determining factor in people's wellbeing and it should not be disregarded, fear of contagion is latent in healthcare professionals and leads to an increase in their levels of stress, anxiety, and fear of providing care to patients.

Regarding the qualitative assessment, three of the analyzed studies have something in common (34–36), namely that the experiences of the COVID-19 outbreak have led to emotional disturbances, which have required coping strategies, not only on a personal, but also on a family and professional level. However, one study, in contrast to the others, included in its conclusions some experiences such as “*But not everything was bad. I have learned a lot*” (35). A Spanish study highlighted that not being single, having a number of years of professional experience and being a man, was associated with a greater use of coping techniques that protect against stressors and threatening emotions. Young people have shown that they have suffered more from isolation from their physical, family and social environment (10). Another issue to highlight is the importance of access to and use of personal protective equipment, as part of the contagion was due to the lack of resources or their misuse (35).

One of the qualitative studies (35), two quantitative studies (23, 32), and one systematic review (40) mention that the latent fear in healthcare workers was that they might infect their family members. This fear sometimes led to self-stigmatization or avoidance behaviors (sleeping in separate beds, not sharing objects/space with family members, among others). In addition, gratitude and appreciation were important issues for most healthcare workers.

On the other hand, most of the quantitative studies, with the exception of one (33), followed a cross-sectional method for measuring and obtaining study results. Among the commonalities between studies, they all shared the assessment of anxiety and stress levels. Also, the most commonly used instruments were the PHQ-9 and GAD-7. Several studies (24, 25, 28) showed associations between anxiety levels and somatic symptoms. Aly et al. (24) indicated that the vast majority of participants suffered from mental health disorders. However, they showed no differences related to age or sex. On the contrary, Chatzittofis et al. (25) and Farì et al. (33) do show in their results significant differences by age and sex, though with opposing results, indicating in the first study that men and older subjects showed increased levels of anxiety while, in the second study, women were more exposed to increased levels of anxiety. In addition, Syamlan et al. (30) indicated that women had a higher prevalence of suffering from depression, anxiety, and stress.

Farì et al. (33) concluded that there were differences in the level of anxiety before and during the pandemic. Only the study by Medeiros et al. (28) mentioned professional efficacy during the pandemic, indicating that just 18% showed low levels of professional efficacy. However, other studies mention that rehabilitation professionals identified negative effects on the quality of services they provided as a consequence of COVID-19 (41, 42). Fear is a human response to threatening situations, and SARS-CoV-2 has become a major global threat, generating this feeling. Emotional burden, perceived risk factors, as well as lack of well-evidenced information, may be associated with the perception of fear of COVID-19 and the impact on health (14).

As for the differences found between types of healthcare professionals, only Szwamel et al. (31) showed that emotional exhaustion was higher in nurses than in the other professionals evaluated, such as physiotherapists. Del Pozo-Herce et al. (14) showed that the pandemic has left a great psychological impact on health professionals, both in terms of stress and in the use of coping strategies, and they indicated that professionals who did not have appropriate working conditions (i.e., type of contract and salary) or those with less years of experience, were more affected in mental health than others.

### Strengths and limitations of study

This study allowed to examine professionals in the area of rehabilitation as an important part of health care during the pandemic. Including this population in the investigation and carrying out research to generate new interventions in mental health are the strongest parts of this research. Likewise, the assessment of rigor and methodological quality of the included studies, and their variables, permit to support solid conclusions and generalizations. Despite the results of interest provided in this research, it would be pertinent to continue deepening the subject of study.

The present study shows some limitations. Firstly, it should be noted that one article written in German was rejected, as no translation could be found, so it is possible that some articles that met the rest of the inclusion criteria were left out for this language reasons. In addition, eleven articles were rejected for not having the exact study population, i.e., only included physicians and nurses but not rehabilitation professionals, or there was not a clear statement about their inclusion in the study. The vast majority of studies was also found to not show strategies to control for confounding factors, except for two articles that do mention this aspect.

Some of the studies did not show a balance between men and women, so it was not possible to assess sex differences related to the variables described in the objective. On the other hand, certain articles did not include a variety of professional groups that would allow establishing differences between professionals/services. Therefore, the findings may have a limited possibility for generalization to all healthcare professionals as the studies only considered physiotherapists to study the professionals of rehabilitation services, and did not include other groups of important professionals, i.e., occupational therapists, speech therapists, etc. Likewise, although there is a variety of countries in the total number of studies, the quantity is not sufficient, and therefore, the representativeness of the results found cannot be extrapolated to the rest of the health professionals who carry out their healthcare work in the rest of the countries of the world.

### Implications and contributions to the field of knowledge

Professionals of the rehabilitation services indicated that the quality of services has been affected by COVID-19, compromising the effectiveness of care (41, 42). For this reason, some activities were temporarily suspended during the last 2 years of the pandemic, and programmes based on work from home were implemented in order to reduce contact with patients. It is therefore relevant to continue researching those factors that compromise the comprehensive care of users in order to implement new care strategies that do not diminish the quality of the service, but allow for continuity.

The use of technology is a good strategy for communication and medical intervention, including rehabilitation. In this sense, it is also important to carry out studies that compare results between professionals in the same health care area, i.e., not only taking into account physiotherapists as the only ones involved in rehabilitation, though this may not be applicable in all cases, as telerehabilitation allows contact to be maintained without fear of contagion. Technology applied to medicine may also empower the patient in their treatment to become an active participant in their recovery, and would also enable the caregiver to assume their role while avoiding overload and being supportive in the process of rehabilitation of the patient, without requiring the continuous presence of the professional in charge.

Equally, the information on the psychological impact of the pandemic throughout the last 2 years contributes to expand knowledge and increases the interest on intervention strategies focused on the health worker's mental health, including professionals in the rehabilitation area. These interventions can be designed to modulate or reduce the risks and consequences of mental health deterioration, as part of a method of prevention of occupational diseases.

## Conclusion

Mental health of healthcare professionals, in general, has been compromised as the COVID-19 pandemic has progressed, compared to before the onset of the pandemic. Women were also found to be more likely to suffer increased levels of anxiety, burnout, and depression, and professionals with children and families showed higher levels of distress and anxiety in caring for patients with COVID-19. Additionally, professionals who were in the front line of the battle against the virus have seen their mental health compromised but with values below those of the general population.

Changes in working hours and care settings, patient overload, fear of becoming infected and infecting loved ones and/or patients, among others, may be precipitating factors for an alteration in the mental health of healthcare professionals in times of the COVID-19 pandemic. Such an alteration can be a major problem at a personal, family, and professional level and can increase the risk of professional malpractice.

## Data availability statement

The original contributions presented in the study are included in the article/Supplementary material, further inquiries can be directed to the corresponding authors.

## Author contributions

Conceptualization: SB-B, RA-C, JG-S, CM-L, JG-I, JF-R, and CR-F. Data curation: SB-B, JG-I, and RA-C. Formal analysis: SB-B, RA-C, JG-S, CM-L, CR-F, JG-I, and CR-F. Investigation: SB-B, RA-C, JG-S, CM-L, JG-I, and JF-R. Methodology: SB-B, JG-S, CM-L, CR-F, and JF-R. Project administration: JG-S and CR-F. Resources: RA-C, JG-S, CM-L, JG-I, and JF-R. Software: SB-B, RA-C, JG-S, and JF-R. Supervision: JG-S, JF-R, JG-I, and CR-F. Validation: RA-C, JG-S, CM-L, and CR-F. Visualization: JF-R and JG-I. Writing—original draft: SB-B, RA-C, JG-I, and JG-S. Writing—review and editing: JG-S, CM-L, JF-R, and CR-F. All authors contributed to the article and approved the submitted version.
