# Supplementary material for: Effects of the COVID-19 pandemic on the mental health of rehabilitation area professionals: A systematic review
Source: Front Public Health. 2022 Dec 8;10:1085820. doi: 10.3389/fpubh.2022.1085820 (PMC9779931; doi:10.3389/fpubh.2022.1085820)
Supplement: Supplementary file 1 [file Table_1.DOCX]

**Table S1. Scores of cross-sectional observational studies** ^(15)^ **(Rehabilitation and COVID-19, Spain, 2022)**

| **Study** | **JBI** | **The participants and the environment are described in detail** | **Inclusion criteria are clearly defined** | **Exposure was validly and reliably measured** | **The criterion used to measure the condition was objective** | **Confounding factors were identified** | **Strategies for dealing with confounding factors** | **Valid and reliable measured results** | **Appropriate statistical analysis was used** |
| --- | --- | --- | --- | --- | --- | --- | --- | --- | --- |
| Alnaser M et al., 2022^(20)^ | 6/8 | ☺ | ☺ | ☺ | ☺ | 😐 | 😐 | ☺ | ☺ |
| Aly H et al., 2021^(21)^ | 7/8 | ☺ | ☺ | ☺ | ☺ | ☺ | ☹ | ☺ | ☺ |
| Chatzittofis A et al., 2021^(22)^ | 7/8 | ☺ | ☺ | ☺ | ☺ | ☺ | ☹ | ☺ | ☺ |
| De Sire A et al., 2021^(23)^ | 6/8 | ☺ | ☺ | ☺ | ☺ | 😐 | 😐 | ☺ | ☺ |
| Jacome C et al., 2021^(24)^ | 6/8 | ☺ | ☺ | ☺ | ☺ | ☹ | 😐 | ☺ | ☺ |
| Medeiros A et al., 2022^(25)^ | 6/8 | ☺ | ☺ | ☺ | ☺ | ☹ | ☹ | ☺ | ☺ |
| Pigati P et al., 2022^(26)^ | 8/8 | ☺ | ☺ | ☺ | ☺ | ☺ | ☺ | ☺ | ☺ |
| Syamlan A et al., 2022^(27)^ | 7/8 | ☺ | ☺ | ☺ | ☺ | ☺ | 😐 | ☺ | ☺ |
| Szwamel K et al., 2022^(28)^ | 8/8 | ☺ | ☺ | ☺ | ☺ | ☺ | ☺ | ☺ | ☺ |
| Yang S et al., 2020^(29)^ | 7/8 | ☺ | ☺ | ☺ | ☺ | ☺ | 😐 | ☺ | ☺ |

Yes: ☺; No: ☹; Unclear or Not applicable: 😐

**Table S2. Scores of analytical observational cohort studies ^(16)^ (Rehabilitation and COVID-19, Spain, 2022)**

| **Study** | **JBI** | **Were the two groups similar and recruited from the same population?** | **Consistency between exposed vs. unexposed measurement** | **Exposure was validly and reliably measured** | **Confounding factors were identified** | **Strategies for dealing with confounding factors** | **Were the groups/participants free of the outcome at the beginning of the study (or at the time of exposure)?** | **Valid and reliable measured results** | **Follow-up time report** | **Follow-up is completed, or reasons for loss are reported** | **Strategies put in place for follow-up** | **Appropriate statistical analysis was used** |  |
| --- | --- | --- | --- | --- | --- | --- | --- | --- | --- | --- | --- | --- | --- |
| Farig G et al., 2022^(30)^ | 7/11 | ☺ | ☺ | ☺ | 😐 | 😐 | 😐 | ☺ | ☺ | ☺ | 😐 | ☺ |  |
| Yes: ☺; No: ☹; Unclear or Not applicable: 😐 | | | | | | | | | | | |  | |

**Table S3. Scores of qualitative studies** ^(17)^ **(Rehabilitation and COVID-19, Spain, 2022)**

| **Study** | **JBI** | **Consistency between stated philosophical perspective and research methodology** | **Consistency between methodology and question/objectives** | **Consistency between the methodology and the method used to collect the data** | **Consistency between methodology and data representation and analysis** | **Consistency between methodology and interpretation of results** | **Cultural and theoretical localisation** | **Influence of the researcher on the sample and vice versa** | **Representativeness of participants** | **Ethical approval by an appropriate body** | **Relationship between findings and data analysis or data interpretation** |
| --- | --- | --- | --- | --- | --- | --- | --- | --- | --- | --- | --- |
| Jeleff M et al., 2022^(31)^ | 7/10 | ☺ | ☺ | ☺ | ☺ | ☺ | 😐 | ☺ | ☹ | ☹ | ☺ |
| Palacios-Cenã, D et al., 2021^(32)^ | 9/10 | ☺ | ☺ | ☺ | ☺ | ☺ | ☺ | ☺ | ☹ | ☺ | ☺ |
| Palacios-Cenã D et al., 2021^(33)^ | 8/10 | ☺ | ☺ | ☺ | ☺ | 😐 | ☺ | ☺ | ☹ | ☺ | ☺ |

Yes: ☺; No: ☹; Unclear or Not applicable: 😐
